# An Uncommon Case of Non‐Specific Interstitial Pneumonia (NSIP) Associated With Idiopathic Hypereosinophilic Syndrome (HES) Reversed by Mepolizumab

**DOI:** 10.1002/rcr2.70270

**Published:** 2025-07-09

**Authors:** Roberto Lipsi, Angelo Coppola, Lorenzo Carriera, Riccardo Moretti, Meridiana Dodaj

**Affiliations:** ^1^ Department of Pulmonology and Sub‐Intensive Respiratory Unit Ospedale Santa Maria della Misericordia Perugia Italy; ^2^ UOC Pneumologia, Ospedale San Filippo Neri‐ASL Roma 1 Rome Italy; ^3^ UniCamillus Saint Camillus International University of Health Sciences Rome Italy; ^4^ Facoltà di Medicina e Chirurgia Università Cattolica del Sacro Cuore Rome Italy; ^5^ Department of Radiology Ospedale Santa Maria della Misericordia Perugia Italy

**Keywords:** Hypereosinophilic syndrome, IL‐5, mepolizumab, non‐specific interstitial pneumonia, pulmonary fibrosis

## Abstract

Hypereosinophilic Syndrome (HES) represents a heterogeneous and complex group of disorders characterised by persistent blood and tissue eosinophilia, leading to progressive tissue damage and organ dysfunction. This spectrum includes both hematologic variants and non‐hematologic forms, either secondary to identifiable causes or idiopathic in nature. In this article, we describe a rare clinical presentation of HES manifesting primarily with pulmonary involvement, diagnosed as Non‐Specific Interstitial Pneumonia (NSIP). Remarkably, the interstitial lung disease showed near‐complete reversibility following targeted inhibition of the IL‐5 pathway with mepolizumab, highlighting the potential role of Th2‐driven eosinophilic inflammation in the pathogenesis of certain forms of interstitial lung disease.

## Graphical abstract

In this article, we describe a rare clinical presentation of Hypereosinophilic Syndrome (HES) manifesting primarily with pulmonary involvement, diagnosed as Non‐Specific Interstitial Pneumonia (NSIP). Remarkably, the interstitial lung disease showed near‐complete reversibility following targeted inhibition of the IL‐5 pathway with mepolizumab, highlighting the potential role of Th2‐driven eosinophilic inflammation in the pathogenesis of certain forms of interstitial lung disease.

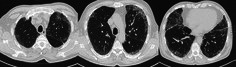

## 1 Introduction

Hypereosinophilic syndromes (HES) are a heterogeneous group of rare disorders characterised by persistent eosinophilia and organ damage due to eosinophilic infiltration. Idiopathic HES (I‐HES) is diagnosed when secondary causes of eosinophilia (parasitic, fungal or mycobacterial infection, allergies, irradiation, toxins, connective tissue diseases or neoplasia) and primary bone marrow disorders have been excluded [1]. Respiratory manifestations are common in HES patients. Eosinophilic infiltration can involve upper and lower airways, as well as alveolar spaces and the lung interstitium [1]. Chest CT findings are variable and generally non‐specific; the most frequently reported patterns include patchy ground‐glass opacities and areas of consolidation. In this article, we describe a rare clinical presentation of HES manifesting primarily with pulmonary involvement, diagnosed as Non‐Specific Interstitial Pneumonia (NSIP) at chest CT scan.

## 2 Case Report

**FIGURE 1:**
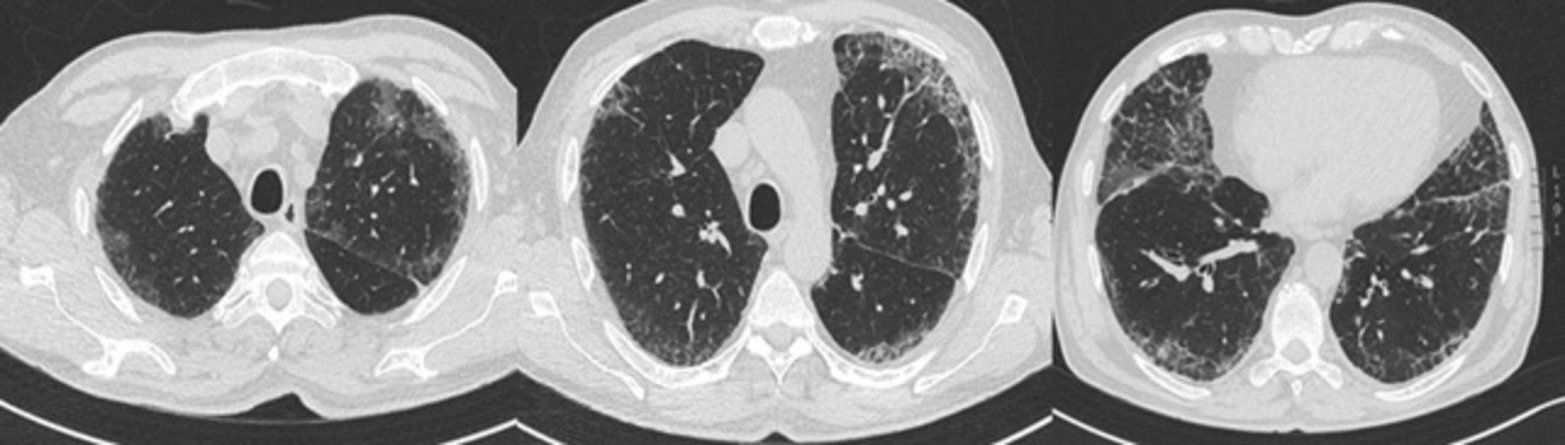
Chest HRCT scan performed at admission. The CT scan revealed bilateral fibrotic interstitial abnormalities associated with ground‐glass opacities and diffuse enlargement of mediastinal lymph nodes.

**FIGURE 2:**
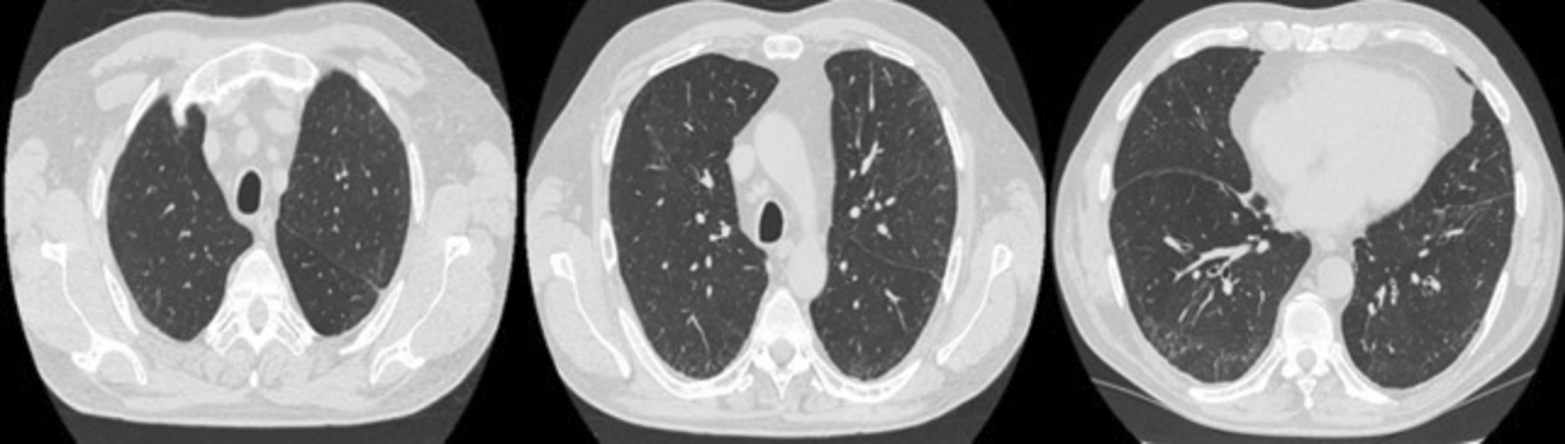
Chest CT performed 6 months after initiating mepolizumab. Near complete resolution of interstitial lung abnormalities 6 months after initiating mepolizumab therapy (some scars areas can be seen in the right lower lobe).

A 71‐year‐old man, a former smoker with a 40 pack‐year history, was referred to our respiratory department for evaluation of acute respiratory failure. He presented with a two‐month history of dry cough and wheezing, accompanied by persistent fever for the past 2 weeks, unresponsive to antibiotic therapy and oral corticosteroids. His past medical history was notable for long‐standing eosinophilic bronchial asthma, managed with inhaled beclometasone/formoterol (100/6 mcg twice daily). He also had gastroesophageal reflux disease and arterial hypertension. No known allergies or occupational exposures were reported. Blood tests revealed a mild inflammation and a significant increase in eosinophil count. There were no signs of renal failure, hepatic failure or electrolyte imbalance. Total IgE and specific inhalant IgE were normal. A high‐resolution chest computed tomography (HRCT) scan revealed bilateral fibrotic interstitial abnormalities associated with ground‐glass opacities and diffuse enlargement of mediastinal lymph nodes (Figure 1). Whole‐body positron emission tomography (PET) confirmed a diffuse increase in fluorodeoxyglucose (FDG) uptake in the mediastinal lymph nodes: upper and lower right paratracheal (SUVmax 6.8), subcarinal (SUVmax 7.05) and bilateral hilar and parahilar regions (SUVmax 5.92). Spirometry revealed a mixed obstructive‐restrictive ventilatory defect, with a severe reduction in the diffusing capacity for carbon monoxide (DLCO). The bronchodilator reversibility test was negative. Given the marked peripheral eosinophilia, in suspicion of Eosinophilic Granulomatosis with Polyangiitis (EGPA), the patient underwent a comprehensive diagnostic workup. The following tests were performed, and all yielded negative results: blood autoantibodies, gastroscopy, rhinoscopy, 24‐h electrocardiogram monitoring and 24‐h urinary protein excretion. Echocardiography showed mild aortic regurgitation, with no significant abnormalities in the remaining valvular structures. Left ventricular ejection fraction and diastolic function were normal. A slight increase in pulmonary artery pressure was detected (PAPs 35 mmHg). No other structural cardiac abnormalities were observed. To exclude parasitic, mycobacterial, or fungal infections, the patient also underwent bronchoscopy with bronchoalveolar lavage (BAL), which did not reveal any evidence of respiratory infection but confirmed an increased eosinophil count (35%). Stool cultures were completely negative. Finally, serum precipitins and tryptase levels were found to be within normal limits. Considering the involvement of both the lung parenchyma and mediastinal lymph nodes, the patient underwent ultrasound‐guided transbronchial needle aspiration (TBNA) of the mediastinal lymph nodes and a lung tissue cryobiopsy. Histological examination of the lymph node sample revealed lymphoid cells with mixed B (CD20^+^) and T (CD3^+^) phenotypes, with a variable proliferative index (Ki67). These were organised into nodular structures consistent with germinal centres, along with histiocytes (CD68/KP1^+^), but without evidence of granulomatous inflammation. Lung biopsy showed homogeneous thickening of the alveolar septa due to the presence of abundant lymphoplasmacytic and eosinophilic inflammatory infiltrate, with mild interstitial fibrosis, Type 2 pneumocyte hyperplasia, and some intra‐alveolar macrophages. Occasional multinucleated histiocytic‐macrophage cells, sometimes forming small aggregates, were also observed, findings compatible with a cellular NSIP pattern. Due to the suspicion of Hypereosinophilic Syndrome (HES), and in order to rule out hematologic malignancies, especially considering the limited diagnostic yield of TBNA in detecting lymphoproliferative disorders, the patient underwent further investigations. These included: lymphocyte phenotyping by flow cytometry (to detect a phenotypically aberrant T‐cell subset), T‐cell receptor (TCR) gene rearrangement analysis in both peripheral blood and bone marrow (to detect T‐cell clonality), and fluorescence in situ hybridisation (FISH) for CHIC2 and FIP1L1‐PDGFRA gene deletions in blood samples. All these investigations returned negative results. The bone marrow examination revealed an eosinophil count of 20% of all nucleated cells, exceeding the threshold considered supportive of HES according to the 2024 WHO and International Consensus Classification [2]. No increase in blasts was observed. The bone marrow biopsy showed no morphological abnormalities, no evidence of dysplasia and no increase in mast cells. Considering that abnormal bone marrow morphology has shown to be a strong indicator of clonal haematopoiesis [3], the absence of such findings, together with the lack of features suggestive of alternative eosinophilic disorders (no history of nasal polyps, no vasculitic complications, ANCA negativity, and no histopathologic features consistent with IgG4‐related disease), further supported the diagnosis of idiopathic HES. The patient was initially treated with intravenous methylprednisolone (1 mg/kg) followed by a gradual tapering with oral prednisone, which led to only a partial response in terms of symptom relief. A follow‐up chest HRCT scan after 6 months of steroid therapy still showed persistent NSIP‐pattern interstitial abnormalities. Consequently, we decided to administer mepolizumab at a dosage of 300 mg per month in addition to oral steroid therapy. This led to a marked reduction in blood eosinophil count, progressive improvement in both symptoms and lung function, and complete discontinuation of corticosteroids. Remarkably, a chest CT scan performed 6 months after initiating mepolizumab showed significant improvement, with near‐complete resolution of the previously documented interstitial lung abnormalities (Figure 2).

## 3 Discussion

This clinical case highlights the diagnostic complexity of I‐HES. The patient had an uncommon presentation with systemic symptoms and interstitial lung disease of the NSIP type, driven by lymphoplasmacytic/eosinophilic inflammation. The first point of interest in this case lies in the identification of an NSIP pattern characterised by a lymphoplasmacytic and eosinophilic inflammatory infiltrate. To our knowledge, this specific histopathological combination has not been previously reported in the literature. The second interesting aspect concerns the resolution of pulmonary fibrosis following treatment with mepolizumab. Pulmonary fibrosis is a chronic, progressive and debilitating lung disease characterised by the accumulation of extracellular matrix (ECM) components within the lung parenchyma, ultimately leading to architectural distortion and impaired gas exchange. Although the direct involvement of IL‐5 in pulmonary fibrosis remains to be fully elucidated, its association with fibrotic mechanisms has become increasingly plausible [4]. Table 1 below summarises key in vitro and in vivo findings supporting a role for IL‐5 in fibrotic lung disease identified through a literature review [5, 6, 7, 8, 9, 10]. We believe that, in our case, the fibrotic process was eosinophil‐driven, and the IL‐5 axis may have contributed to the development of an NSIP‐type fibrotic pattern. The complete clinical remission and sustained radiological improvement observed with mepolizumab suggest that IL‐5 blockade may have interrupted a Th2‐driven profibrotic pathway in this form of interstitial lung disease. Further evidence is needed to confirm these findings and to better define the efficacy of mepolizumab in this context.

**TABLE 1:** Summary of key studies supporting a role for IL‐5 in pulmonary fibrosis.

Study	Model/Approach	Main findings	Implications
Luo et al. [5]	In vitro eosinophil stimulation	IL‐5 enhances eosinophil responsiveness to TGF‐β, by priming eosinophils to express higher levels of TGF‐β receptors. TGF‐β stimulation led eosinophils to express extracellular matrix (ECM) proteins	Synergistic profibrotic effect via the IL‐5/TGF‐β/SMAD signalling axis
Gharaee‐Kermani et al. [6]	Bleomycin‐induced pulmonary fibrosis in mice + anti‐IL‐5 antibody treatment	Anti‐IL‐5 reduced lung eosinophilia, cytokine expression (including TGF‐β, MCP‐1), and fibrosis	IL‐5 promotes fibrosis via eosinophil recruitment and fibrogenic cytokines upregulation; targeting IL‐5 reduces fibrotic burden
Huaux et al. [7]	IL‐5 knockout mice with bleomycin‐induced fibrosis	IL‐5 knockout mice were not protected; however, high IL‐5 levels worsened fibrosis	IL‐5 may not initiate fibrosis but amplifies fibrotic progression; excessive IL‐5 is detrimental
Bajbouj et al. [8]	Human bronchial fibroblasts and lung tissue (asthmatics vs. non‐asthmatics).	IL‐5Rα expression upregulated in asthmatic fibroblasts and epithelium	IL‐5 responsiveness is enhanced in certain fibrotic or inflammatory conditions
Chen et al. [9]	In vitro and in vivo pulmonary fibrosis models	IL‐5Rα upregulation linked to EMT and ECM production via JAK2/STAT3 activation	IL‐5 pathway drives EMT and fibrogenesis beyond eosinophils
Flood‐Page et al. [10]	Endobronchial biopsies in asthmatic patients treated with Mepolizumab	Reduced expression of ECM proteins (tenascin, lumican, procollagen III)	IL‐5 inhibition can reduce ECM deposition

Abbreviations: ECM, Extra Cellular Matrix; EMT, Epithelial‐Mesenchymal Transition.

## Author Contributions

Conceptualization: R.L. and M.D. Data collection: R.L. Writing – original draft preparation: R.L., A.C., L.C., R.M. and M.D. Writing – review and editing: R.L., A.C., L.C., R.M. and M.D. Supervision: R.L. All authors have read and agreed to the published version of the manuscript.

## Ethics Statement

The authors declare that written informed consent was obtained for the publication of this manuscript and accompanying images using the consent form provided by the Journal.

## Conflicts of Interest

The authors declare no conflicts of interest.

## Data Availability

The data that support the findings of this study are available from the corresponding author upon reasonable request.
